# Organic chemistry meets polymers, nanoscience, therapeutics and diagnostics

**DOI:** 10.3762/bjoc.12.161

**Published:** 2016-08-02

**Authors:** Vincent M Rotello

**Affiliations:** 1 Department of Chemistry, University of Massachusetts-Amherst, 710 North Pleasant Street, Amherst, Massachusetts 01003, USA

**Keywords:** organic synthesis, supramolecular, nanoparticle sensing

## Abstract

The atom-by-atom control provided by synthetic organic chemistry presents a means of generating new functional nanomaterials with great precision. Bringing together these two very disparate skill sets is, however, quite uncommon. This autobiographical review provides some insight into how my program evolved, as well as giving some idea of where we are going.

## Review

### My roots – synthetic organic

My interest in chemistry built on my incessant tinkering. When I was young, I was the sort that would take things apart to see how they worked. I also put them back together, occasionally with no extra parts… Eventually, the ability to create overcame the desire to dissect, and I started pursuing photography. My efforts in this domain progressed until I started considering art as a career. Alongside this artistic pathway, however, I developed an interest in chemistry. What intrigued me at the start was connectivity – how atoms could be strung together. I explored both art and photography at Illinois Institute of Technology, but science and scientists ended up deciding me. It started with Chem. 237, Organic Chemistry. This course was taught by Pete Johnson, who introduced me to retrosynthetic analysis, and in the process showed me how I could achieve the connectivity I had doodled when younger. This classroom work was followed up soon thereafter by practical training at the hands of Phil Garner. Phil was an old school 'guts and glory' synthetic type, and I learned from him everything I needed to about the practical side of synthetic organic. This work culminated in the first paper I co-authored [1], where I learned painful lessons with the Fieser triangle at the twilight of the pen and ink era.

With my belly full of synthetic fire, I sought out a place to pursue my trade. In the mid-80s Yale University was hotbed for synthesis, and picking out an advisor was a difficult choice. I ultimately chose Harry Wasserman, based on the freedom he gave his group in choosing and attacking synthetic and physical challenges. During this period I developed and finished a variety of syntheses. My break came, however, when looking in the back pages of the journal *Heterocycles* in the "New Natural Products" section. I noticed a rather interesting new molecule known as rapamycin that had the vicinal tricarbonyl motif in vogue in the Wasserman group. When I suggested we go after this molecule, Harry demurred, pointing out its complexity and that "nobody would be interested". I kept my eye on the literature, however, and when Dave Williams published the "Central America" fragment of the related macrolide FK-506, I urged Harry to see if he could get some intermediate that I could use to demonstrate our methodology for tricarbonyl synthesis. Dave generously obliged by sending ~200 mg of an advanced intermediate. With hands shaking, I cracked the vial and started on the synthesis. Working with small-scale reactions (<1 mg), I eventually worked out a high-yielding synthesis of the Williams fragment [2]. This synthesis generated some buzz, catching the eye of folks like Sam Danishefsky and facilitating my next move.

### Moving to Legoland

Sorting out what I wanted to do after grad school was a bit of a challenge. In those days I knew I wanted to be an academic, but what I wanted to do scientifically was an open question. I started thinking about proposals for postdoctoral fellowships, but the synthetic ideas I generated didn't fire me up like I thought they should. I really enjoyed the power of organic synthesis, but I wanted to do something with the molecules that I laboriously fashioned. Once again my love of connectivity kicked in, this time with supramolecular chemistry. I started thinking of molecules instead of atoms as building blocks. I looked around for professors with a like mind, and applied to Julius Rebek at Pittsburgh. Between when I applied and when I joined as an NSF postdoctoral fellow Julius had moved to MIT for his brief stay in the Boston area before heading off to Scripps.

While in the Rebek group I used my synthetic abilities while gaining the insight into physical organic chemistry that has informed the rest of my career. I started out working on self-replicating systems [3], developing new systems that had novel capabilities, including external regulation [4]. I also took on a brutal project, focused on the synthesis of water-soluble analogs of Kemp's triacid. This project was a massive effort, with a huge number of reactions required to optimize the initial steps. We did, however, obtain the desired receptors and observe some interesting binding processes in water [5]. During this part of my time in the group, Julius offered that if I stayed an additional year I could work on projects of my own. During this time I discovered my inner mentor. Much to the annoyance of my labmates, I built a veritable army of undergraduates, pursuing supramolecular chemistry, along with a project in fullerenes [6–7].

**Figure 1:**
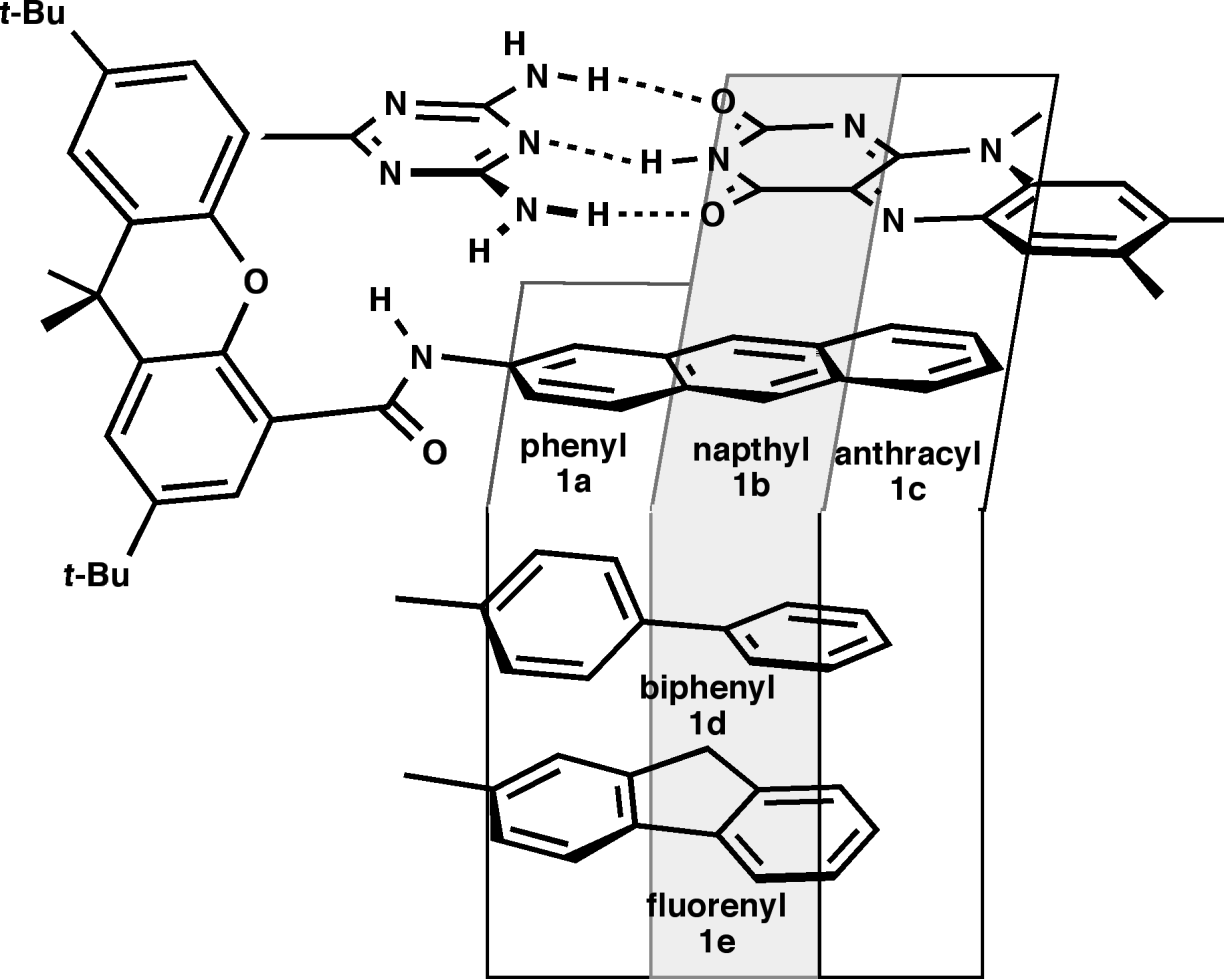
Flavoenzyme model system for determining the role of aromatic stacking in flavin redox processes. Reprinted with permission from [10]. Copyright (1997) American Chemical Society.

After finishing my postdoctoral work, I moved to the University of Massachusetts. I chose UMass based on its quality and longstanding reputation for collaborative research. Upon arrival, I collected a fired up group of graduate students and went to it. I maintained my fullerenes project [8], and initiated a set of three other supramolecular projects. Of these projects, our work on flavoenzyme models was the one that really took off. Using electrochemistry, we were able to gain a real understanding of how forces such as hydrogen bonding [9], aromatic stacking (Figure 1) [10] and donor atom–π interactions [11] influence the energetics of redox processes. We also used integrated experimental and computational techniques to actually establish what hydrogen bonding does to the electrons in hydrogen-bonded complexes [12]. Finally, we put this all together to generate molecular switches and devices [13]. This work has continued through our long productive collaboration with Graeme Cooke, now at University of Glasgow, moving from redox-active systems [14–15] to photovoltaics (along with Ifor Samuel at St. Andrew’s) [16].

### Go big, go nano

**Figure 2:**
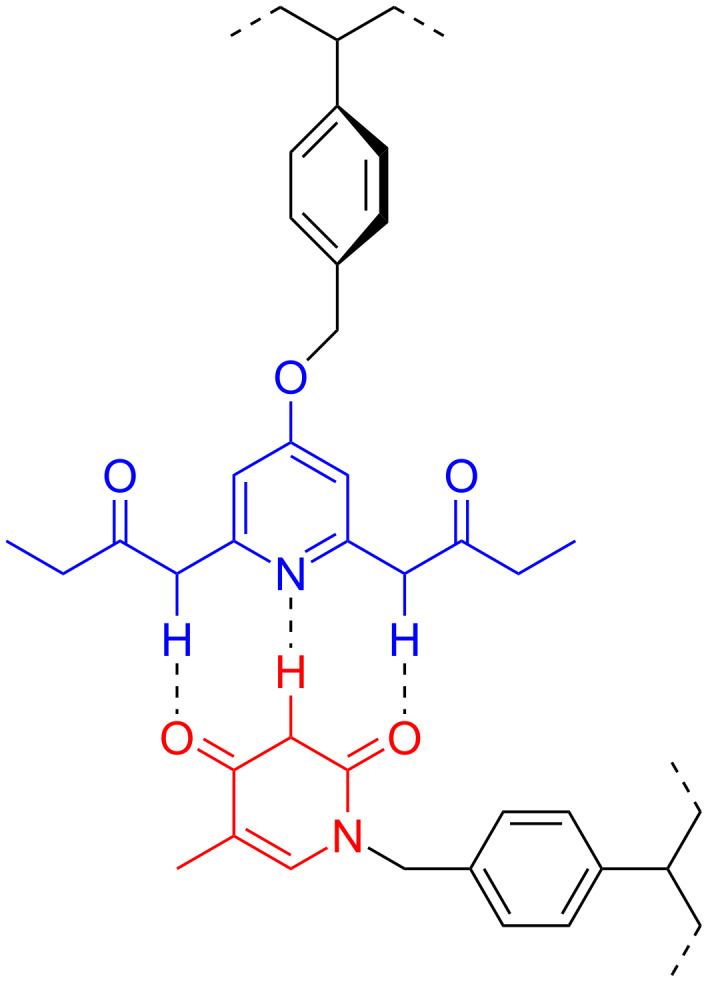
Recognition element-functionalized polymers for 'plug and play' modification and self-assembly.

After four or so years of work on redox-modulated recognition two things happened. First, I realized that we could predict what would happen with the systems before we made them. This took much of the fun out of the work. Simultaneously, I received tenure, and tried to sort out what I wanted to do for my next career phase. Our next move was into polymers, where we started the conceptual journey we are still taking. The key question we asked is "we know what happens when you have one host–guest dyad, but what happens when you have 10, 50, 100 on a polymer?" On a straightforward level, we were able to demonstrate that we could use non-covalent sidechain modification between multivalent polymers and monovalent guests to generate "plug and play polymers" (Figure 2) [17]. We also showed that we could self-assemble a polymer around an electroactive guest, effectively encapsulating and isolating it [18].

The research really became interesting when we started mixing multivalent complementary polymers together. When we mixed together diaminopyridine and uracil polymers together in chloroform we generated a turbid solution. Under the microscope we found that the turbidity surprisingly arose from vesicular structures [19]. Through quite a bit of experimentation we determined that the unprecedented self-assembly process was driven by self-sorting of the polymer chains to provide vesicle walls with denser recognition elements in the middle than at the outside [20].

While we were working with polymers, we were just starting to move into nanoparticles. As with the polymers, we started off studying the interactions of recognition element-functionalized nanoparticles with monovalent guests – in this case our old friend flavin where we showed modulation of the flavin redox potentials [21]. Taking this research one step further, we created a nanoparticle with a mixed monolayer consisting of hydrogen bonding and aromatic staking sidechains. When we incubated this NP with flavin we observed an increase in binding over time, i.e., we were able to template the particle to the guest [22]. We have since demonstrated this templation with peptides [23] and are (still!) trying to definitively show templation to proteins.

**Figure 3:**
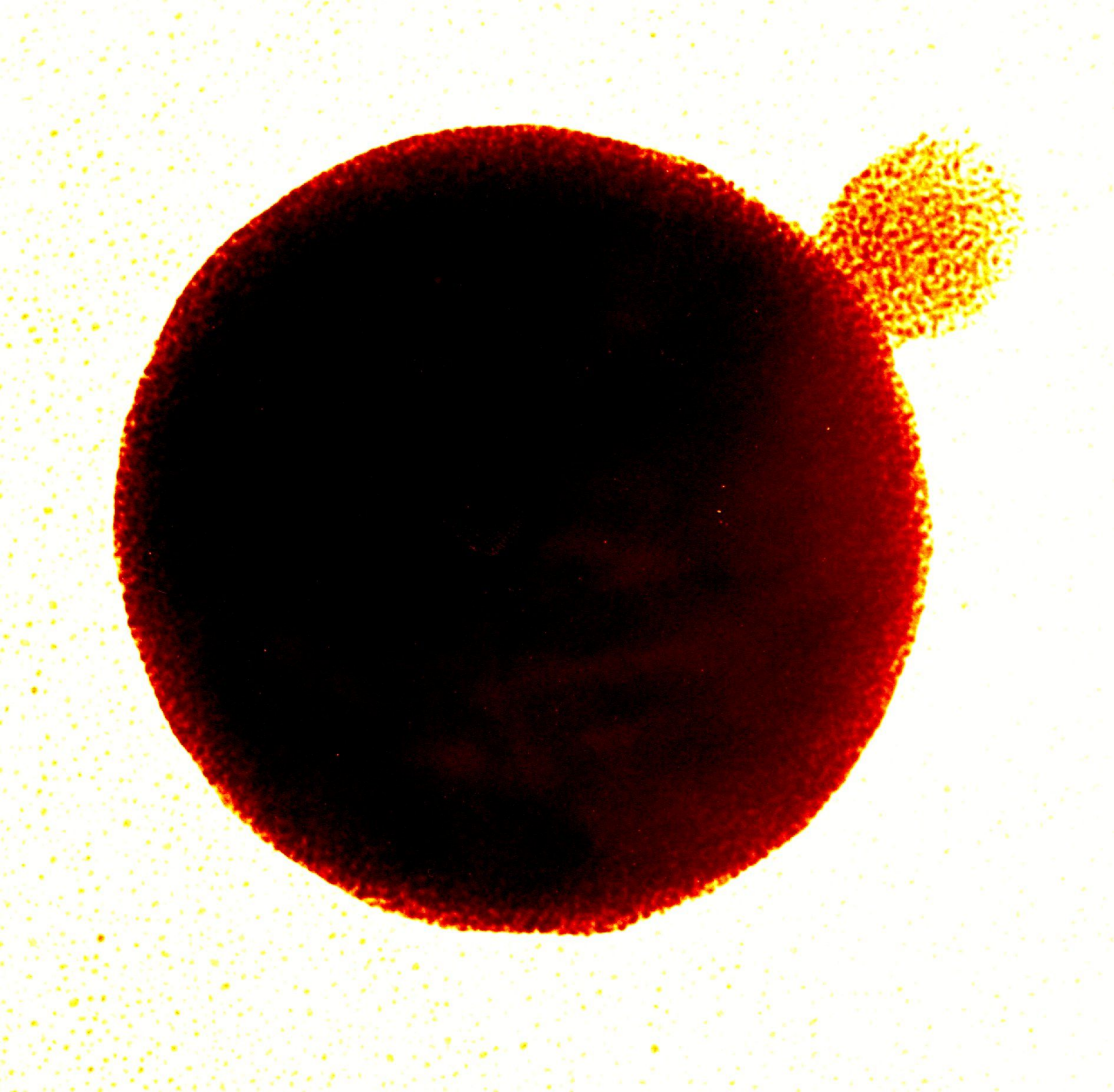
Recognition-mediated assembly of nanoparticle–polymer constructs. Reproduced from [24].

As I mentioned above, I am an incessant tinkerer, a trait that has rubbed off on the group. When students mixed complementary versions of the polymers and nanoparticles described above, we were quite surprised to find that we generated regular spherical and network structures (Figure 3) [24]. This "bricks and mortar" assembly process provides a modular system where structure and stoichiometry of the components drives structure formation. These assemblies set us on a path of generating nanocomposite materials, including regular structures using diblock copolymers [25–26] and nanoparticle–protein [27–28] and nanoparticle–nucleic acid composites [29].

Concurrently with our 3D self-assembly, we pursued the use of nanoparticles for surface modification. This research has focused on the use of these particles to efficiently impart functional properties to surfaces [30]. including anti-fouling properties [31]. When combined with nanoimprint lithography [32] this process gives us access to nano-textured nanopatterned surfaces [33–34]. We have recently employed this strategy to control cell growth on surfaces [35], including using the surface properties of the nanoparticle to dictate cell selectivity [36].

### Nanoparticles meet biology

Our move into nanocomposites coincided with our efforts to interface materials and biology – the current core focus of the lab. We started off looking at nucleic acids, where we showed that cationic nanoparticles could bind to anionic DNA and inhibit transcription [37]. We also developed a number of strategies for delivery of small molecules, including glutathione-mediated release of covalently attached thiols [38], as the effective release of drugs adsorbed into the cationic monolayers of nanoparticles [39], and even photoactivatable drug [40] and DNA release [41]. When we started looking at how particles interact with proteins, however, very little was known about how these materials would play together. We found out pretty quickly that the answer was "not very well". Binding of the nanoparticle induced protein denaturation, with loss of bioactivity [42]. We hypothesized that this denaturation arose from interaction of the protein with the hydrophobic elements of the simple ligands we were using. This hypothesis led us to create what we call the "tabula rasa" ligand [43], namely a ligand that features a hydrophobic interior for self-assembly, and a short tetra(ethylene glycol) layer to block interactions of the hydrophobic interiors with proteins [44]. These particles were indeed "blank" (but not as blank as our later zwitterionic “corona-free’ particles [45]), and behaved like high molecular weight poly(ethylene glycol) [46]. Once we appended simple anionic and cationic recognition elements to the surface these system bound proteins with high affinity [47] and some degree of selectivity [48]. What was surprising is that not only did the particle–protein binding process not denature the proteins, it actually stabilizes them [46]! This result stills surprises researchers who follow the dogma that proteins must denature at interfaces. One of the other observations we made using isothermal titration calorimetry is that nanoparticle–protein interactions using the tabula rasa-based particles mimicked the thermodynamics of protein–protein interactions quite well [49].

Once we were able to have proteins and nanoparticles work in harmony [50] we started working on designing systems with emergent behavior, i.e., where the particle–protein complex behaves differently than either of the two components. A prime example of this synergy is when we showed that nanoparticle–enzyme complexes showed altered substrate selectivity [51], with the particle dictating the enzyme kinetics by acting as a "filter" for substrate and product [47]. Another area where we demonstrated synergy is in the area of Pickering emulsions. These emulsions are made by interfacial assembly of particles at oil–water interfaces. We showed that nanoparticles and proteins could be self-assembled at this interface [52], retaining their activity. When the oil core was crosslinked, these systems worked even better, enhancing enzyme activity [53], even under extreme chemical and thermal conditions [54].

As we were learning the rules for nanoparticle-biological interactions we started looking into applications for these self-assembled materials. Our first real success came when we demonstrated very efficient DNA transfection (i.e., gene delivery) using gold nanoparticles [55]. In later work we improved on our gene delivery vehicles [56], demonstrated the delivery of siRNA [57] and enzymes [58] using nanoparticle assemblies. All of these systems (as well as essentially all of the other examples of nanomaterial delivery vehicles in the literature) occurred via endosomal uptake. The problem with this route is that what goes into the endosome tends to stay in the endosome, and eventually be degraded. Since most of the interesting things in cells require access to the cytosol (including materials destined to the nucleus), this entrapment is a major limitation [59].

One of the beauties of supramolecular chemistry is its modularity. We started looking into the use of the Pickering emulsions described above for delivery applications. There was a challenge: nobody (including us) could generate emulsions with diameters small enough (<200 nm) for use as in vivo delivery vehicles [60]. Once again, supramolecular chemistry came to the rescue. Anslyn showed that guanidinium groups bound strongly to carboxylates [61]. This led to the surmise that this interaction could be used to "pin" arginine-capped nanoparticles to oil droplets comprised of fatty acids. This trick worked remarkably well, providing ~150 nm nanocapsules. These capsules were unstable in serum however. Reaching back to our nanocomposite work, we were able to use bricks and mortar assembly of anionic proteins (transferrin) and cationic nanoparticles to create stable capsules [62]. These capsules delivered hydrophobic drugs and dyes to cells very effectively. A puzzling question arose however: dyes were delivered into cells much faster than the particles on the outside of the capsule. Clearly, endosomal uptake would result in identical rates of uptake, leading us to surmise that uptake occurred through a membrane fusion process.

**Figure 4:**
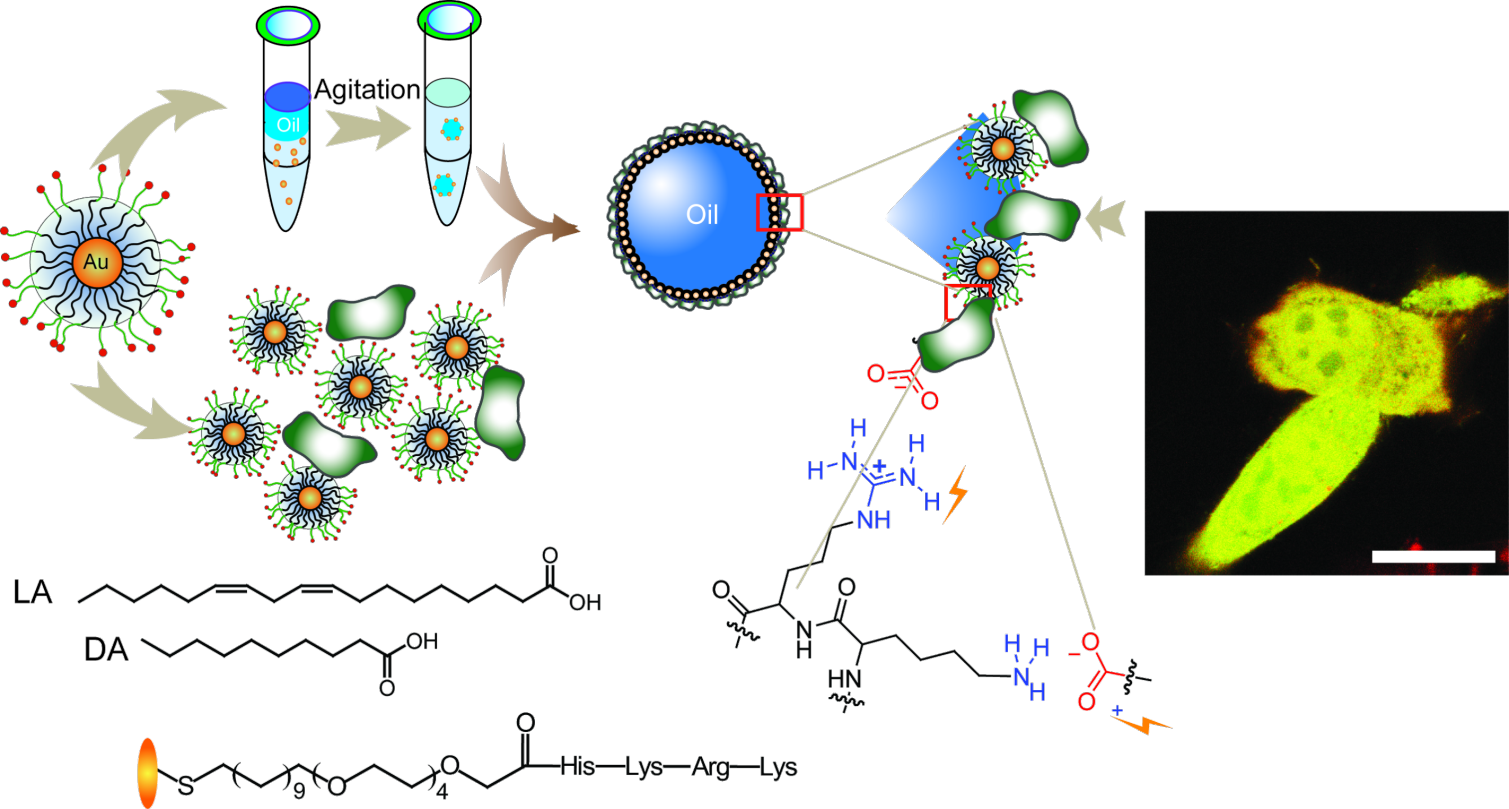
Cytosolic delivery of GFP to cells using nanoparticle-stabilized nanocapsules. Adapted with permission from [63]. Copyright (2013) American Chemical Society.

Driven by the desire to deliver biological payloads directly to the cytosol, we tested our system for the very challenging goal of protein delivery using green fluorescent protein (GFP). It worked even better than we hoped, with complete cytosolar distribution of the GFP observed (Figure 4) [63]. This ability to "dump" proteins into cells is unprecedented, allowing us to deliver proteins capable of intracellular localization – the next frontier of targeting [64]. We also made use of the oil interior of the capsule to provide dual protein (caspase 3) and therapeutic (paclitaxel) delivery where the two payloads worked synergistically for chemotherapy [65]. Being supramolecular types, we figured we could swap out the anionic proteins used above for anionic siRNA [66]. In this case we were right – we can deliver siRNA directly into the cytosol with great efficiency [67].

Concurrent with our delivery efforts, we were working on ways to track nanoparticles in living systems. Back in 2008 I had a student working jointly with Richard Vachet, and I suggested the rather crazy notion of taking some cells, putting in nanoparticles, and then throwing them in the matrix-assisted laser desorption ionization (MALDI) mass spectrometer without matrix. Quite surprisingly, all the student could see was the ligand on the particle – with essentially no signal from all of the rest of the stuff found in cells [68]. This was the start of a collaboration that continues to this day, where we have used laser desorption ionization (LDI) to characterize ligands on particles [69–70], supramolecular chemistry in cells [71], track NPs in fish [72] and plants [73], and to image NPs in animal organs – after shooting them into mice [74]. More recently, we bought an inductively coupled plasma (ICP) mass spectrometer that allows us to quantify elements. We have used ICP together with LDI to determine the stability of quantum dots [75] and gold nanoparticles in cells [76]. Once again, a tribute to doing the occasional eccentric experiment.

### Building nanonoses

As we were developing our delivery vehicle platforms, we were starting to think about sensing applications. One of the things that we observed in our protein binding work was that our nanoparticles had varying affinities for different proteins [77]. This differential affinity made our particles potential sensor elements for employing the "chemical nose" strategy to proteins. The challenge was that particles bound to proteins look quite similar to particles that aren't. Clearly, we needed a means of transducing protein binding. The breakthrough occurred at the Fall 2006 ACS meeting where the ever-charming Uwe Bunz and I had the simultaneous inspiration of using Uwe's highly fluorescent poly(phenylene ethynelene) "molecular wire" polymers as transducers. These polymers would be quenched by the gold core of the nanoparticles while bound, regaining fluorescence after being displaced by protein analytes. This strategy worked quite well, allowing us to discriminate proteins at considerably lower concentration than prior studies [78]. Using the supramolecular chemist's ability to oversimplify, we soon extended these studies to bacterial [79] and mammalian cells [80] under the pretext that cell surfaces are "complex solutions" confined to surfaces [81].

While Uwe's polymers were incredibly useful, they had two limitations. The first limitation is that they tended to aggregate and self-quench in complex media, wiping out the signal. This aggregation became an issue when we attempted to sense changes in serum profiles. We overcame this issue using Nature's fluorescent polymers, namely fluorescent proteins. Using gold nanoparticles and green fluorescent protein we were able to rapidly detect very small changes in serum protein profiles in undiluted serum [82] a project that we are currently testing in human studies. I should point out that we have managed to marry the strenths of the polymer and fluorescent protein systems [83] using polymer–protein FRET to provide modulated output [84].

**Figure 5:**
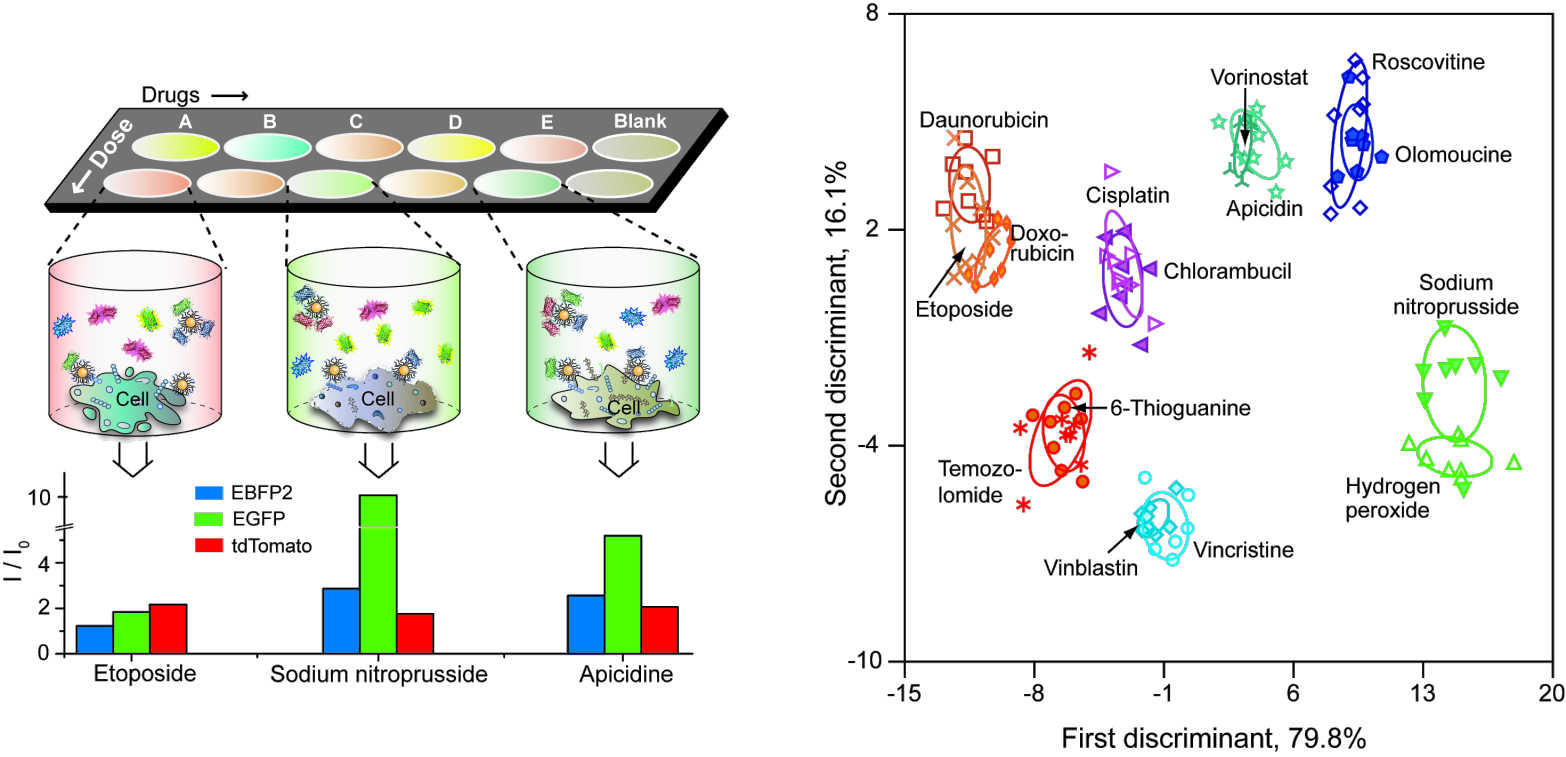
Rapid determination of therapeutic mechanisms using three-channel nanoparticle fluorescent protein nanosensors. Adapted from [85].

You'll remember that I said that conjugated polymers had two limitations. The second issue we had was lack of choices in emission color – blue and green are easy, yellow is challenging, and getting a red conjugated polymer with a respectable quantum yield is nigh impossible. We had an idea, however, that we could use multi-channel sensing to increase our sensor capabilities, with the goal of creating "one well" chemical nose sensors. To this end we expressed ourselves some red, green and blue fluorescent proteins and set to work on cell surface sensing. Rather than going after something easy, we decided to see if we could discriminate mechanisms of chemotherapeutics. This project worked better than we could have hoped, with complete discrimination of eight different mechanisms, all in the matter of minutes (Figure 5) [85]. The sensor was tested against therapeutics and was able to identify the mechanisms of new drugs in the training set, and equally importantly identify if the therapeutic acted by a mechanism novel to the training set. The capabilities of this sensor are quite impressive, but you may ask "what is it on the cell surface that the sensor is responding too?" We have an excellent clue to that question: changes in glycosylation (through mutation or glycolosis) generate very strong sensor responses, implicating that our sensor responds to changes in glycosylation [86] which is probably also the mechanism in play for our successful determination of biofilms using this platform [87].

### Whither next?

You can probably tell from the above ramble that predicting where I am going next is an incredibly challenging question for me. We have a number of areas we are currently exploring, including the creation of nanoparticle-based antimicrobials [88–89] building on our studies showing that nanoparticles are effective against antibiotic-resistant bacteria such as MRSA [90] and nanocapsule systems work against the biofilms [91] made by these nasties [92]. We have also made a foray into bioorthogonal chemistry, using nanoparticles to encapsulate transition metal catalysts, generating “nanozymes” that can catalyze processes in cells that even the cells can’t do [93]. We have even been doing some immunology [94], including turning on the innate immune system [95] and figuring out what macrophages like to eat [96].

We have plenty to work on just taking our systems where they need to go, be it in vivo or in the clinic [97–98]. Broader picture, what I really want to see is the integration of the synthetic and biological worlds to generate new systems that can do things that neither can do alone [99–100]. This vision of "cyborg" constructs with emergent behavior is motivating both my own research and my editorial efforts with *Bioconjugate Chemistry*. I feel that this is an area where chemistry can really impact the human condition, of course keeping in mind the cautionary tales provided by many Sci-Fi novels and movies.

### Something about the author

Like anyone with a job, I live two lives. As you can probably surmise, science moves me at work. As I travel, I also enjoy the comradeship of scientists around the world, and can become quite evangelical about the roles the scientific community can serve to bridge gulfs between nations and cultures. To be honest, I also enjoy seeing the world and its many wonders, reveling in both the "big" sights and running at dawn in a new town. And I have been accused of traveling on my stomach – I do have a fondness for food .

At home, I am a family man (if not a Family Guy…). Coming from the school that food equals love, my love of cooking is likewise a major factor in my life. The skills I developed as a synthetic chemist give me an understanding of ingredients and techniques that allows me to cook cuisines from around the world. Fortunately my wife and I are inveterate exercisers, and my son's metabolism is still rapid enough to avoid (excessive) weight gain. This exercise is typically done with our rather neurotic Weimaraner Trudy, allowing us to get double duty from our toil.
